# The symptom burden of Irritable Bowel Syndrome in tertiary care during the COVID‐19 pandemic

**DOI:** 10.1111/nmo.14347

**Published:** 2022-03-03

**Authors:** Hithin Noble, Syed Shariq Hasan, Peter J. Whorwell, Dipesh H. Vasant

**Affiliations:** ^1^ Neurogastroenterology Unit Wythenshawe Hospital Manchester University NHS Foundation Trust Manchester UK; ^2^ Division of Diabetes Endocrinology and Gastroenterology University of Manchester Manchester UK

**Keywords:** COVID‐19 pandemic, disorders of gut‐brain interaction, Irritable Bowel Syndrome

## Abstract

**Background:**

The COVID‐19 pandemic caused unprecedented disruption to healthcare services worldwide with well‐documented detrimental effects on mental health. Patients with refractory disorders of gut‐brain interaction such as Irritable Bowel Syndrome (IBS) seen in tertiary care tend to exhibit higher levels of psychological comorbidity, but the impact of the pandemic on IBS symptom severity in tertiary care is unknown.

**Methods:**

As part of routine clinical care, consecutive tertiary referrals with refractory IBS patients prospectively completed a series of baseline questionnaires including IBS symptom severity score (IBS‐SSS), non‐colonic symptom score, Hospital Anxiety and Depression (HAD), and Illness impact scores. The symptom severity questionnaire data were compared for consecutive patients seen in tertiary care 12 months before and after the onset of COVID‐19 pandemic restrictions.

**Key Results:**

Of 190 consecutive tertiary referrals with IBS, those seen during the pandemic had greater IBS severity (IBS‐SSS: 352 vs. 318, *p* = 0.03), more severe extra‐intestinal symptoms (non‐colonic score: 269 vs. 225, *p* = 0.03), sleep difficulties (*p* = 0.03), helplessness and loss of control (*p* = 0.02), but similar HAD‐Anxiety (*p* = 0.96) and HAD‐Depression (*p* = 0.84) scores. During the pandemic, unmarried patients (*p* = 0.03), and keyworkers (*p* = 0.0038) had greater IBS severity.

**Conclusions and Inferences:**

This study has shown for the first time that patients seen in tertiary care with refractory IBS during the COVID‐19 pandemic had a significantly higher symptom burden emphasizing the importance of gut‐brain axis in IBS. Furthermore, lack of support and perceived loss of control appear to be contributory factors.

## Graphical abstract

Consecutive patients with Irritable Bowel Syndrome assessed in a tertiary center during the first 12‐months of COVID‐19 pandemic restrictions had significantly greater gastrointestinal and extra‐intestinal symptom severities compared to those seen during the 12‐months before the pandemic.

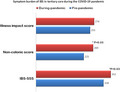

**Key Points**

- Patients with refractory Irritable Bowel Syndrome seen in tertiary care had a greater severity of gastrointestinal and extra‐intestinal symptoms during COVID‐19 pandemic restrictions.
- Clinical anxiety was high, and sleep disturbance, a feeling of loss of control, and isolation appeared to be contributory factors to the greater Irritable Bowel Syndrome severity seen during the pandemic.
- This study emphasizes the role of the gut‐brain axis and the need for access to multidisciplinary integrated care within a biopsychosocial model for Irritable Bowel Syndrome during the recovery phase post‐pandemic.

## 1 INTRODUCTION

In recent years, significant advances in the understanding of Irritable Bowel Syndrome (IBS) include its reclassification as a disorder of gut‐brain interaction (DGBI),^1^ and the recognition of the importance of a spectrum of gastrointestinal, extra‐intestinal and psychological symptom clusters in the identification of subgroups.^2^ Recent data suggest that these symptom clusters have long‐term prognostic significance.^3^ Those with a high psychological symptom burden at baseline have higher symptom severity, are more likely to be refractory to treatments, more likely to seek consultations,^4^ and are more likely to be referred to tertiary care.

The COVID‐19 pandemic has caused significant disruption to healthcare services. Moreover, the impact of the subsequent lockdowns and social distancing regulations on mental health are well documented.^5^ In the aftermath of the COVID‐19 pandemic, much of the focus on recovery of gastroenterology services has been on restoring endoscopic activities, and services for “high‐risk” chronic gastrointestinal conditions including inflammatory bowel and liver diseases.^6^ Despite patients with IBS having risk factors for symptom regression via the gut‐brain axis, nothing is known about the impact of the pandemic on IBS severity and the burden that this could place on healthcare resources.

The aims of this study were therefore to compare prospectively obtained data on baseline gastrointestinal, extra‐intestinal and psychological symptom severities in consecutive patients with refractory IBS seen in tertiary care during the COVID‐19 pandemic restrictions, with patients seen over the same time period before the onset of the pandemic.

## 2 MATERIALS AND METHODS

### 2.1 Patient population

As part of their routine care, consecutive patients with IBS referred to a tertiary DGBI center prospectively completed a series of baseline symptom questionnaires. All patients fulfilled clinical diagnostic criteria for IBS^7^ verified by a gastroenterologist and all had failed to respond to IBS dietary and medical treatments for 12 months, and were eligible for consideration for gut‐brain psychological therapies as per United Kingdom national recommendations.^7^ Prior to being allocated a clinic appointment, all patients prospectively completed the following baseline questionnaires in paper form; Bristol stool chart to determine IBS‐subtypes as per Rome IV criteria – diarrhea predominant (IBS‐D), constipation predominant (IBS‐C), mixed (IBS‐M) and unclassified (IBS‐U),^8^ IBS symptom severity score (IBS‐SSS),^9^ non‐colonic symptom score,^10^ Hospital Anxiety and Depression (HAD),^11^ and illness impact questionnaire as a measure of quality‐of‐life.^10^

### 2.2 Outcome measures

#### 2.2.1 Irritable Bowel Syndrome Severity Score (IBS‐SSS)

The IBS‐SSS score allows the evaluation of the severity of abdominal pain and distension, frequency of pain, bowel habit satisfaction, and how patients perceive IBS interferes with their life on a visual analog scale of 0–100.^9^ The maximum possible score is 500, with severe IBS indicated by scores of >300. This scoring system is now universally used in IBS studies and trials to measure IBS severity and assess response to therapeutic interventions.

#### 2.2.2 Non‐colonic symptom score

The IBS non‐colonic score assesses the severity of extra‐intestinal symptoms. Patients are required to score each component using a visual analog scale of 0 to 100.^12^ These components include nausea and vomiting, early satiety, headaches, backaches, lethargy, excess flatulence, heartburn, urinary symptoms, thigh pain, and muscle and joint pains. To obtain the final non‐colonic score, the sum of the 10 component sub‐scores is divided by two. The maximum score that can be obtained is 500, with higher scores illustrating a worse extra‐intestinal clinical picture.^12^ This questionnaire has been routinely used to evaluate extra‐intestinal symptom outcomes in patients with IBS in a number of our studies.^10^, ^12^, ^13^, ^14^, ^15^

#### 2.2.3 Hospital anxiety and depression questionnaire

The HAD Questionnaire is an established questionnaire widely used to determine the levels of anxiety and depression experienced by patients.^11^ The questionnaire has 14 components, scored from 0 to 3, with seven relating to anxiety and seven to depression. The maximum score for either depression or anxiety is 21. Scores of below 8 are considered normal, while scores ≥8 indicate clinical depression or anxiety.^11^

#### 2.2.4 Illness impact questionnaire

The illness impact score has an inverse relationship to a patient's quality life, with 15 components on a visual analog scale, scored out of 500.^12^ A higher illness impact score illustrates a poorer patient quality of life. For instance, this includes evaluating feelings of irritability, inferiority or hopelessness to asking patients to rate the enjoyment of their leisure time.

### 2.3 Data collection and analysis

Demographic and questionnaire data were analyzed and compared between patients that completed pre‐clinic questionnaires in the 12 months before the COVID‐19 pandemic (22/03/2019–22/03/2020), with those that completed their pre‐clinic baseline questionnaires during 12 months of COVID‐19 pandemic restrictions affecting the Greater Manchester region, UK between 23/03/2020 and 23/03/2021.

During the post‐pandemic period studied, there were national or regional restrictions in place throughout the 12 months in accordance with UK national government and public health policies. This study period encompassed three national lockdowns, and at both the beginning (23/03/2020), and end of the post‐pandemic study period (23/03/2021), citizens were required by law to “stay at home,” with significant restrictions on non‐essential gatherings, other than “keyworkers” in certain professions critical to the pandemic response (health and social care, education and childcare, key public services, local and national government, food and other necessary goods, transport, public safety and national security, and utilities, communication and financial services) citizens were all required to work from home, and non‐essential travel and non‐essential retail outlets remained closed.^16^

The study period post‐onset of the COVID‐19 pandemic included a period between 23/03/2020 and 03/06/2020 when all non‐emergency/non‐urgent outpatient care and diagnostics including the DGBI clinic were suspended, due to redeployment of medical staff to emergency, acute care and urgent cancer referrals.

Due to the restrictions that were in place throughout the post‐pandemic period, routine face‐to‐face clinics did not resume during the timeframe of the study. All patients that completed their pre‐clinic questionnaires after resumption of the clinic therefore had their tertiary clinic appointment remotely, via video consultation. As per the DGBI clinic's normal pre‐pandemic procedures, following resumption of the DGBI clinic on 03/06/2020, all patients returned pre‐clinic questionnaires in paper form, via the postal system, prior to being allocated their video consultation appointment.

Questionnaire data were analyzed using descriptive statistics and pre and post‐pandemic data were compared using the Chi‐square and Mann–Whitney *U*‐test where appropriate on a standard statistical software package (Stats Direct v.3.1.1, United Kingdom). *p*‐values ≤0.05 were considered statistically significant.

## 3 RESULTS

190 consecutive tertiary referrals with IBS were included, 107 patients were assessed in the 12 months prior to the pandemic, and 83 patients completed their pre‐clinic questionnaires during the 12‐months post‐onset of COVID‐19 restrictions in the UK. There were no significant differences in the demographics of the two cohorts, Table 1.

**TABLE 1:** Demographics of tertiary referrals with IBS seen 12 months before compared with those seen during the COVID‐19 pandemic

Patient characteristics	Pre‐pandemic cohort (*n* = 107)	Pandemic cohort (*n* = 83)	*p*‐value (95% CI)
Median age, years (IQR)	45 (25)	40 (21)	*u* = 5112.5, *p* = 0.10 (−1 to 9)
Gender, females (%)	84 (79)	71 (86)	*x* ^2^ = 1.1, *p* = 0.29 (0.3 to 1.3)
IBS sub‐type: (number, %)			
IBS‐D	31 (28.9)	16 (19.3)	*x* ^2^ = 1.9, *p* = 0.17 (0.9 to 3.4)
IBS‐C	41 (38.3)	37 (44.6)	*x* ^2^ = 0.5, *p* = 0.47 (0.3 to 1.6)
IBS‐M	34 (31.8)	30 (36.1)	*x* ^2^ = 0.2, *p* = 0.63 (0.4 to 1.5)
IBS‐U	1 (0.9)	0 (0)	N/A
Marital status: (number, %)			
Married	48 (44.9)	27 (32.5)	*x* ^2^ = 2.5, *p* = 0.12 (0.9 to 3.1)
Unmarried	51 (47.6)	47 (56.6)	*x* ^2^ = 1.2, *p* = 0.28 (0.4 to 1.2)
Divorced/widowed	7 (6.5)	9 (10.8)	*x* ^2^ = 0.6, *p* = 0.43 (0.2 to 1.6)
Unknown	1 (0.9)	0 (0)	N/A

### 3.1 Symptom severity

**FIGURE 1:**
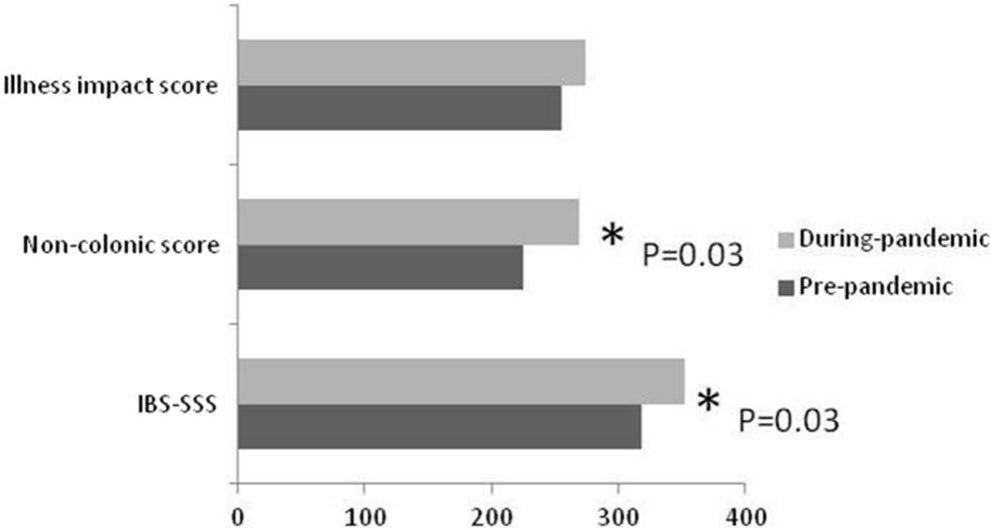
Differences in median gastrointestinal symptom severity (IBS‐SSS), extra‐intestinal symptom severity (non‐colonic score) and illness impact scores in patients referred to a tertiary IBS clinic in the 12 months before and during COVID‐19 restrictions (*p < 0.05)

Patients with refractory IBS assessed in tertiary care during COVID‐19 restrictions had higher IBS‐SSS (*p* = 0.03) with higher abdominal pain (*p* = 0.05) and distension (*p* = 0.008) sub‐scores, and higher overall extra‐intestinal symptom burden (*p* = 0.03), Figure 1, Tables 2 and 3.

**TABLE 2:** Comparison of median sub‐scores of IBS symptom severity in tertiary patients with IBS in the 12 months before and after the COVID‐19 pandemic (*p ≤ 0.05)

Median IBS‐severity sub‐score (maximum score 100)	Pre‐pandemic cohort	Pandemic cohort	*p*‐value (95% CI)
Abdominal pain severity	50	63	*u* = 3246, *p* = 0.05* (−16 to 0)
Abdominal pain frequency	70	80	*u* = 3556, *p* = 0.30 (−15 to 0)
Abdominal distension severity	60	75	*u* = 3008, *p* = 0.008* (−18 to −1)
Dissatisfaction with bowel habit	75	75	*u* = 3586, *p* = 0.36 (−9 to 2)
Interference with life	80	83	*u* = 3333, *p* = 0.09 (−9 to 0)
Total median IBS‐SSS (maximum score 500)	318	352	*u* = 3644, *p* = 0.03* (−56 to −3)

**TABLE 3:** Comparison of median extra‐intestinal symptom scores in referrals with IBS 12 months before and after onset of the COVID‐19 pandemic (*p ≤ 0.05)

Median non‐colonic sub‐scores (max. score 100)	Pre‐pandemic cohort	Pandemic cohort	*p*‐value (95% CI)
Nausea/vomiting	26	44	*u* = 3464, *p* = 0.13 (−18 to 0)
Early satiety	49	37	*u* = 4219, *p* = 0.51(−4 to 14)
Headaches	46	50	*u* = 3603, *p* = 0.26 (−16 to 3)
Backache	59	68	*u* = 3564, *p* = 0.22 (−16 to 2)
Lethargy	82	83	*u* = 3817, *p* = 0.62 (−6 to 4)
Excess wind	75	75	*u* = 3781, *p* = 0.73 (−10 to 4)
Heartburn	25	25	*u* = 3819, *p* = 0.69 (−11 to 4)
Urinary symptoms	50	64	*u* = 3367, *p* = 0.07 (−22 to 0)
Thigh pain	10	25	*u* = 3717, *p* = 0.43 (−8 to 0)
Musculoskeletal aches and pains	53	64	*u* = 3737, *p* = 0.47 (−14 to 4)
Median total non‐colonic scores (max. score 500^∆^)	225	269	*u* = 3649, *p* = 0.03* (95% CI: −55 to −2)

When comparing differences between IBS‐subtypes, median IBS‐SSS was numerically higher in all IBS‐subtypes during the pandemic cohort, but this difference was most marked in IBS‐D (pre‐pandemic vs. pandemic: IBS‐C 309 vs. 359, *u* = 705, *p* = 0.12 (95% CI:‐83 to 10); IBS‐M 313 vs. 345, *u* = 549, *p* = 0.16 (95%CI:); IBS‐D 323 vs. 379, *u* = 250, *p* = 0.04 (95% CI: −98 to −3)).

Unmarried patients seen during the pandemic had a higher median IBS‐SSS compared to their unmarried counterparts seen prior to the pandemic (pre‐pandemic vs. during pandemic: unmarried patients 320 vs. 359, *u* = 897, *p* = 0.03 (95% CI: −75 to −3); married patients 296 vs. 352, *u* = 504, *p* = 0.15 (95%CI: −75 to 16)).

Within the pandemic cohort, those with IBS who worked within keyworker occupations defined by the UK government as critical to the response to the pandemic (*n* = 33), had significantly greater IBS severity when compared to those that were either able to work from home, those that were retired, or unemployed (*n* = 50); median IBS‐SSS keyworkers versus non‐keyworkers: 335 vs. 278.5, *u* = 1,133, *p* = 0.0038 (95% CI: 19 to 87).

### 3.2 Illness impact, anxiety and depression scores

The overall illness impact of refractory IBS on quality‐of‐life was similar in both groups, but sleep disturbance (*p* = 0.03), helplessness and loss of control feelings (*p* = 0.02) were significantly higher in those seen during the pandemic (Table 4). Anxiety levels were similarly high in both refractory IBS cohorts, with clinical levels of anxiety (HAD‐A ≥ 8) in 87/107 (81%) in the pre‐pandemic group and (63/83) 76% in the group treated during the pandemic (*X* ^2^ = 0.82, *p* = 0.36 (95% CI: 0.68 to 2.77)). Similarly, clinical levels of depression (HADS ≥ 8) were common in both cohorts (pre‐pandemic 53/107 (49%) vs. during pandemic 41/83 (49%), *X* ^2^ = 0.0003, *p* = 0.98 (95% CI: 0.56 to 1.78)). There was also no difference in median anxiety and depression scores between the two cohorts (HAD‐Anxiety: 11 vs. 11.5, *u* = 4184, *p* = 0.96 (95% CI: −1 to 1); HAD‐Depression: 8 vs. 8, *u* = 4021, *p* = 0.84 (95%CI: −2 to 1)).

**TABLE 4:** Comparison of median IBS illness impact scores in tertiary referrals with IBS 12 months before and during the COVID‐19 pandemic (*p ≤ 0.05)

Median Illness impact sub‐scores (max. 100)	Pre‐pandemic cohort	Pandemic cohort	*p*‐value (95% CI)
Coping with problems	62	50	*u* = 4180, *p* = 0.34 (−2 to 11)
Confidence and security	66	73	*u* = 4180, *p* = 0.86 (−9 to 7)
Quality of sleep	53	68	*u* = 3130, *p* = 0.03* (−18 to 0)
Irritability	58	53	*u* = 3622, *p* = 0.48 (−10 to 4)
Frequency of worrying	75	76	*u* = 3589, *p* = 0.42 (−11 to 3)
Enjoyment of life	50	56	*u* = 3432, *p* = 0.20 (−14 to 1)
Feelings of hopefulness	50	59	*u* = 3430, *p* = 0.20 (−15 to 2)
Physical well‐being	54	68	*u* = 3438, *p* = 0.21 (−13 to 2)
Relationships with others	28	32	*u* = 3889, *p* = 0.93 (−7 to 7)
Maintaining friendships	25	25	*u* = 3792, *p* = 0.84 (−8 to 6)
Inferiority	50	50	*u* = 3723, *p* = 0.68 (−13 to 7)
Feeling wanted	31	50	*u* = 3449, *p* = 0.22 (−18 to 1)
Helplessness and lack of control	50	70	*u* = 3086, *p* = 0.02* (−20 to 0)
Difficulty in making decisions	50	50	*u* = 3939, *p* = 0.82 (−7 to 9)
Total illness impact score (max. score 500^±^)	255	274	*u* = 3845, *p* = 0.18 (−13 to 6)

## 4 DISCUSSION

This study is the first to demonstrate changes in the symptom profiles and severity of IBS referrals to tertiary care during the COVID‐19 pandemic. The findings of our study are likely to have important clinical consequences. There is evidence that medication consumption, healthcare utilization and hospitalizations have increased in patients with DGBIs including IBS during the COVID‐19 pandemic.^17^ Therefore, our findings of significantly higher IBS symptom severity and higher extra‐intestinal symptom burden, during the pandemic are likely to have significant implications for resource utilization in already stretched healthcare systems.

These findings are unlikely to be by chance as the pre‐pandemic data in our study on gastrointestinal, extra‐intestinal, and psychological symptom scores (Table 2), are almost identical to published baseline data using the same questionnaires from almost 1,500 patients with refractory IBS from our unit which have been stable over the past 10 years.^10^, ^14^, ^15^ Moreover, the observed reciprocal relationship between higher perceived abdominal distension severity and abdominal pain severities in the pandemic group is consistent with the recent literature on IBS symptom severity.^18^

The patients in this tertiary, refractory, population had high levels of psychological comorbidity with the majority of patients in both the pre‐pandemic and pandemic cohorts having clinical levels of anxiety. It was interesting to note that there was little difference in the anxiety and depression scores between the two groups before and during the pandemic, especially with regard to anxiety. Similar to our findings in a population of tertiary patients with IBS who had high levels of anxiety and depression even in the pre‐pandemic group, longitudinal studies that have followed up patients who had pre‐existing high levels of anxiety and depression pre‐pandemic have found minimal changes in the symptom severity levels of anxiety and depression during the COVID‐19 pandemic.^19^, ^20^ This therefore suggests that other psychological factors, beyond anxiety and depression, relating to their response to the pandemic might be driving their symptom deterioration. The effects of stress on the gut‐brain axis and how this contributes to symptoms in DGBI is well recognized. Recent evidence suggests that stress has resulted in an increase in the prevalence of IBS during the COVID‐19 pandemic.^21^ Moreover, patients with IBS and high levels of anxiety, such as those included in our study are more likely to be susceptible to severe exacerbations due to aberrant coping strategies^22^, ^23^, ^24^, ^25^ and lower levels of resilience,^26^ catastrophizing and somatization,^27^ potentially explaining the more severe somatic (non‐colonic) symptoms, extreme loss of control and helplessness as well as sleep disturbance among those within the pandemic cohort in our study. While the exact reasons for more sleep disturbance in the pandemic group is unclear, social support during the pandemic restrictions may be a contributory factor. During the COVID‐19 pandemic there is evidence that lower levels of social support were associated with higher risk of sleep disturbance and psychological effects.^28^ Interestingly, our data also suggest that social support may be a protective factor. Those that were unmarried had higher IBS symptom severity. Social isolation, which has been shown to be associated with gastrointestinal symptoms and related psychological distress during the pandemic,^29^ is a possible explanation, although unfortunately it was not possible to draw firm conclusions on this retrospectively, due to the lack of data available on whether or not those who were unmarried, lived alone. During the pandemic, occupation also appeared to be an important factor associated with IBS symptom severity. Compared to keyworkers who were critical for the response to the pandemic, those that could stay at home (non‐keyworkers, unemployed and retired) had significantly less severe IBS. This is consistent with the findings from a study in France where 21.3% of patients reported an improvement in IBS symptoms during COVID‐19 restrictions presumed to relate to improved toilet access while working from home.^23^

During recovery of gastroenterology services following the COVID‐19 pandemic, understandably much of the focus has been on recovery of services for patients with “high‐risk” and life limiting organic conditions. Restoration of endoscopic activities, cancer diagnostics, liver diseases and inflammatory bowel diseases have had to be prioritized. However, despite not being a life limiting condition, IBS is a highly prevalent condition worldwide^30^ associated with significant healthcare utilization and economic costs.^31^ Recent expert reviews and guidelines on severe, difficult to treat and refractory IBS have all advocated best management within a biopsychosocial framework emphasizing integrated multidisciplinary care including access to medical, dietary and psychological/ behavioral therapies.^7^, ^32^, ^33^ Indeed, there is evidence that investment in this form of multidisciplinary treatment can be extremely efficacious and cost effective compared to standard models of gastroenterology care.^34^, ^35^, ^36^ However, despite the high prevalence of IBS, even before the pandemic, there are relatively fewer highly specialized tertiary centers with the resources to offer integrated multidisciplinary care for patients with severe refractory IBS. These tertiary services are now likely to be under increasing pressure following the pandemic for several reasons. Firstly, many existing tertiary neurogastroenterology services had resources diverted into the acute pandemic response,^37^ and many institutes may now be reluctant to re‐invest in “low‐risk,” chronic conditions such as IBS, during the current climate and recovery phase. Secondly, our data have demonstrated higher than usual symptom severity in patients with already refractory IBS in tertiary care. This suggests that specialist tertiary services need to be equipped to provide multidisciplinary care to prevent unnecessary hospital admissions, and related healthcare costs at a time when healthcare services are already under extreme pressures. Another challenge faced by neurogastroenterologists is the necessity to find new ways of working in the aftermath of the pandemic to meet this demand. Most of the integrated multidisciplinary care provided prior to the pandemic in tertiary IBS care has traditionally been provided face‐to‐face. There is increasing recognition within the field of DGBI of the importance of enhanced communication in optimizing patient‐provider interactions to achieve positive outcomes,^38^ and it is therefore possible that COVID‐19 enforced changes to the ways in which patients access and experience healthcare could have contributed to the observed findings in the post‐pandemic group. The pandemic has however provided new opportunities for innovative delivery of care remotely via video‐consultations,^13^, ^39^ remote helplines^40^ and by the use of group therapy^41^ to widen access, and future studies should evaluate their long‐term efficacy after the pandemic.

There are several limitations to our study. Due to suspension of the DGBI clinic during the acute phase of the pandemic response, the group sizes are unequal with fewer tertiary patients with IBS seen during the 12 months of the pandemic restrictions. Secondly, data were not available on the COVID‐19 infection status of the patients included. It is therefore not possible to determine whether COVID‐19 infections could have contributed to our symptom severity results, particularly in the IBS‐D group which has previously been shown to be associated with COVID‐19 infection.^17^ Due to the pandemic constraints, we also cannot eliminate that the threshold for referral to tertiary care may have been higher resulting in only the most severe cases being seen. Nonetheless, the disruptions resulting in reduced capacity within our DGBI clinic during the acute phase of the pandemic are not unique to our center and have been reported in other countries.^37^ The findings are therefore likely to be applicable to other centers with important implications for service recovery. Despite reduced clinical capacity and priority for neurogastroenterology services in the post‐COVID era, referral rates with DGBIs have remained high throughout the pandemic. Previous studies have reported an increasing prevalence of IBS during the pandemic,^21^ increasing DGBI related healthcare costs and hospitalizations,^17^ and this makes our findings even more relevant as the demands and waiting lists within tertiary DGBI care are expected to increase even further with the expected wave of post‐infectious IBS related to COVID‐19 infection itself.^42^

In conclusion, patients seen in tertiary care with IBS during the pandemic had a significantly higher symptom burden emphasizing the importance of gut‐brain axis in IBS. Furthermore, lack of support and perceived loss of control appear to be contributory factors. These observations suggest that investment and provision of integrated multidisciplinary IBS care within a biopsychosocial model should not be ignored when planning the recovery of gastroenterology services.

## CONFLICT OF INTEREST

None of the authors have any relevant conflicts of interests to declare.

## AUTHOR CONTRIBUTIONS

Hithin Noble collected and analyzed data, and helped draft the manuscript, Syed Shariq Hasan helped with data collection and analysis and reviewed the manuscript, Peter J. Whorwell reviewed the manuscript and provided important intellectual input, Dipesh H. Vasant conceived and designed the study, helped analyze data and write the manuscript and is the guarantor of this article.
